# T Cell Recognition of Autoantigens in Human Type 1 Diabetes: Clinical Perspectives

**DOI:** 10.1155/2011/513210

**Published:** 2011-07-19

**Authors:** Roberto Mallone, Vedran Brezar, Christian Boitard

**Affiliations:** ^1^INSERM, U986, Hôpital St Vincent de Paul, 82 avenue Denfert Rochereau, 75014 Paris, France; ^2^Université Paris Descartes, 75005 Paris, France; ^3^Hôpital Cochin-Hôtel Dieu, Assistance Publique des Hôpitaux de Paris, 75679 Paris, France

## Abstract

Type 1 diabetes (T1D) is an autoimmune disease driven by the activation of lymphocytes against pancreatic **β**-cells. Among **β**-cell autoantigens, preproinsulin has been ascribed a key role in the T1D process. The successive steps that control the activation of autoreactive lymphocytes have been extensively studied in animal models of T1D, but remains ill defined in man. In man, T lymphocytes, especially CD8^+^ T cells, are predominant within insulitis. Developing T-cell assays in diabetes autoimmunity is, thus, a major challenge. It is expected to help defining autoantigens and epitopes that drive the disease process, to pinpoint key functional features of epitope-specific T lymphocytes along the natural history of diabetes and to pave the way towards therapeutic strategies to induce immune tolerance to **β**-cells. New T-cell technologies will allow defining autoreactive T-cell differentiation programs and characterizing autoimmune responses in comparison with physiologically appropriate immune responses. This may prove instrumental in the discovery of immune correlates of efficacy in clinical trials.

## 1. Introduction

Type 1 diabetes (T1D) is an autoimmune disease driven by the activation of lymphocytes against pancreatic *β*-cells. While the successive steps that control the activation of autoreactive lymphocytes have been extensively studied in animal models, the disease process remains ill defined in the human [1]. However, the predominant role of T lymphocytes is characteristic of both mouse and human T1D. In the mouse, T1D is transferred into naive recipients by T cells, is prevented by antibodies that target T lymphocyte activation, and fails to develop when key genes in T lymphocyte differentiation or activation are non functional [2]. T1D is a highly multigenic disease both in the mouse [3] and in the human [4]. In man, T lymphocytes, especially CD8^+^ T cells, are predominant within insulitis in most [5–11], although not all [12], observations. Occurrence of T1D in a patient deprived of B lymphocytes further underscores the role of T lymphocytes [13]. 

Remarkable features in human T1D are the long preclinical phase that precedes the development of full-blown hyperglycemia [14] and the high recurrence level of autoimmunity in long-standing patients who have been treated with exogenous insulin for years [15]. The first detection of autoantibodies can occur at any time during life. However, it peaks at one to three years of age in a large subset of children who develop early T1D. A second incidence peak is seen around puberty and show more heterogeneous autoantibody profiles than in early forms of T1D [14]. Rapid diabetes recurrence is seen in T1D recipients of an isograft from a discordant, nondiabetic twin and is accompanied by an almost exclusive CD8^+^ T-cell islet infiltration [15]. It, thus, seems that *β*-cell-specific T lymphocytes maintain immune memory for years after T1D onset. However, differentiation patterns of autoreactive T lymphocytes, once diabetes diagnosed, remain largely unknown.

Among *β*-cell autoantigens (Table 1), proinsulin has been ascribed a key role in the T1D process [1, 16]. In the NOD mouse, injection of insulin-specific T-cell clones accelerates diabetes and protection is obtained by injecting insulin in incomplete Freund's adjuvant in prediabetic mice [2, 17]. Altered diabetes development in proinsulin 1^−/−^ or 2^−/−^ NOD mice makes a strong case for the primary role of insulin in this model [18–20]. By contrast, deficient expression of glutamate decarboxylase (GAD) or islet antigen 2 (IA-2) has no striking effect on diabetes development in this model [21, 22]. Antigen spreading may thus explain the activation of T cells against a long list of autoantigens once the autoimmune process on. T cell clones that are specific for GAD, chromogranin and islet-glucose-6-phosphatase catalytic subunit-related protein (IGRP) are indeed detected and transfer diabetes into naive NOD recipients [23–26]. In man, insulin and proinsulin are common targets of autoantibodies and T cells in (pre)diabetic individuals [27–37]. Insulin autoantibodies (IAA) are the first to be detected in children at risk for T1D and carry a high positive predictive value for diabetes in siblings of T1D patients [14, 28]. However, autoantibodies and T cells have been detected against autoantigens other than insulin in the human [38].

The immune image of the *β*-cell is that of native components of the *β*-cell membrane in their three-dimensional conformation, as seen by B lymphocytes, and, more importantly, of fragments of intracellular *β*-cell proteins in the form of 8–11 mer peptides loaded onto class I major histocompatibility complex (MHC) molecules, as seen on the *β*-cell surface by CD8^+^ T lymphocytes. In addition, professional antigen-presenting cells (APCs) present fragments of autoantigens that are phagocytosed following the release of subcellular *β*-cell particles or *β*-cell debris in the extracellular milieu and loaded onto MHC class I and class II molecules. Given the key role of T lymphocytes in the T1D disease process, the cartography of autoantigen-derived peptides that are presented to class I-restricted CD8^+^ T cells and class II-restricted CD4^+^ T cells, although still incomplete, will be the main focus of this paper.

## 2. T Cell Recognition of Insulin

Both direct evidence in the mouse and indirect evidence in the human point at insulin as a key autoantigen in T1D autoimmunity. The search for T cell recognition of insulin and the characterization of insulin epitopes recognized by T lymphocytes along disease development is thus a major challenge.

### 2.1. CD4^+^ T Cell Responses to Proinsulin

The study of T cell responses to *β*-cell autoantigens have long been limited to MHC class II-restricted responses but have faced major methodological caveats, precluding translation into routine laboratory procedures. CD4^+^ T cell responses to exogenous insulin have first been studied [39]. They have been shown to be exacerbated in response to inhaled insulin in T1D patients and in patients treated with insulin analogs, in particular insulin detemir. They are beyond the scope of this paper that will focus on T cell responses to insulin as part of the autoimmune response to *β*-cells. Autoimmunity to insulin seen in the rare insulin autoimmune syndrome in patients with Grave's disease will not be detailed either [39]. Following studies associating the detection of antibodies to exogenous insulin to HLA-DR4, HLA-DR4-restricted T cell responses have first been prioritized. The characterization of the high susceptibility DQ8 molecule led to the characterization of DQ8-restricted responses in addition to DR4-restricted responses.

Proliferative T cell responses have been reported in the human against both insulin and proinsulin, especially in recent-onset T1D patients and prediabetic individuals [29–31, 35] although also in nondiabetic subjects in some reports [36, 40]. Despite treatment with insulin, long-standing T1D patients were often found low responders [30, 31], as also observed in case of CD8^+^ T cells [32]. An inverse correlation has been observed between the presence of anti-insulin autoantibodies and T cell responses to proinsulin [29, 30], although not to insulin [40] and not in all studies [40, 41]. Some patients showed a response to proinsulin, although not to insulin, indicating that C-peptide residues were among the epitopes recognized [30]. Responses to insulin have been observed in 25% of T1D patients and 10% of siblings in a model in which proliferative responses were increased in siblings of T1D patients, although not T1D patients themselves, by addition of anti-DQ antibodies, implying the presence of primed suppressive HLA-DQ-restricted T cell responses to insulin in siblings [42]. 

Epitopes of proinsulin have been characterized in the human [43] (Table 2). Using class II knockout mice that were transgenic for the DQ8 diabetes susceptibility class II allele, two immunodominant preproinsulin regions have been defined, spanning residues 1–24 and 44–63, respectively. Immunodominant regions, that is, 14–33 and 74–93, were different in diabetes resistant transgenic mice that express the diabetes resistant DQ6 allele [44]. Epitopes spanning the C-peptide and A-chain junction have also been reported as immunodominant in DR1*0401 transgenic mice [45]. Using a preproinsulin peptide library, immunodominant epitopes located within the C peptide (C_13-29_) and B chain (B_11-27_) were preferentially recognized by CD4^+^ T cells from autoantibody positive individuals at high risk for T1D development whereas CD4^+^ T cells from insulin-treated T1D patients were responsive to native insulin and insulin B chain (B_1-16_). Unexpectedly, an IL-4 and IL-10 response was predominant in both the naive CD45A and memory CD45RO T cell compartments [36]. 

The role of the insulin B chain peptide B_9-23_ in the NOD mouse led to test whether this peptide could also be recognized in human T1D. Importantly, the three dimensional structure of the DQ8 molecule complexed with insulin peptide B_9-23_ has been determined [46]. Short-term T cell lines obtained following a 7–10 day incubation of peripheral blood mononuclear cells (PMBCs) from 10/12 recent-onset T1D patients, while not from controls, have been shown highly proliferative to B_9-23_. Insulin B_9-23_-specific T cell lines were restricted by HLA-DQ8 which shows striking structural similarities with the NOD mouse IA^g7^ class II molecule. Substantial numbers of interferon *γ*-producing cells were detected in most recent-onset T1D patients and prediabetic subjects using an ELISpot assay [34]. However, the extent to which a B_9-23_ proliferative assay can apply to routine evaluation in T1D remains elusive. Presentation of peptide B_9-23_ was confirmed in class II knock out mice that were made transgenic for the human DQ8 allele [47]. The characterization of peptides eluted from HLA-DR4 class II molecules further allowed defining naturally processed proinsulin epitopes that clustered in the C peptide and C peptide-A chain junction. Significant responses were observed against C_13-32_, C_19_-A_3_ and C_22_-A_5_. A positive interferon *γ* response to proinsulin peptides was detected in 56% of 25 T1D patients and in none of 14 control subjects. By contrast, an IL-10 response to proinsulin peptides was detected in one out of four control subjects and T1D patients. An inverse correlation was observed between the interferon *γ* and the IL-10 response to IA-2 and proinsulin peptides, although not to tetanus toxoid, in patients and control subjects. Type 1 diabetic patients who showed an IL-10 response were older at onset of diabetes than patients who showed an interferon *γ* response [48].

In a different set of experiments, expansion of T cells from pancreatic draining lymph nodes of subjects with T1D and controls allowed characterizing T cell clones. While T cell clones from control individuals were highly polyclonal in light of heterogeneous V*β* T cell receptor usage, around 50% of T cell clones from 2 of 3 T1D patients expressed identical V*β* chains, favoring an antigen-driven expansion of T cells. Half of clonally expanded clones from the 2 patients were specific for insulin A_1-15_. Both patients were HLA-DR401 which is strongly associated with susceptibility to T1D, but also to insulin antibodies in insulin-treated patients. Both patients, however, were long-standing T1D subjects who had been treated with insulin for over than 10 years when lymph node were collected. No response to insulin in blood, spleen or pancreatic lymph nodes from a type 2 diabetic patient treated with insulin was observed. It is hypothesized that T cells residing in the pancreatic lymph nodes may persist in individuals in whom *β* cells have been eliminated for years [49]. The use of a human DR4B1*0401-restricted CD4^+^ T cell clone that was obtained from a prediabetic, insulin autoantibody-positive child further allowed showing that posttranslational modifications of insulin epitopes impacts on recognition by autoreactive T cells. The T cell clone was specific for A_1-13_ and proliferated to human islet insulin, indicating that the epitope was derived from native insulin. The authors failed to obtain CD4^+^ T cell clones specific for this epitope from two healthy donors. Recognition by the A_1-13_-specific T cell clone was dependent on the formation of a disulfide bond between adjacent cysteine residues A_6_ and A_7_ which, however, did not alter peptide binding to HLA-DR4. The A_6_ and A_7_ cysteine residues were required for T cell recognition by this clone, while the A_11_ cysteine residue was not. Recognition of A_1-13_ was dependent on the presence of oxidized residues that allowed formation of a disulfide bond between residues A_6_ and A_7_ [50]. These data strengthen previous evidence that the oxidation state of insulin-derived peptides plays a role in recognition by insulin A_1-14_-specific T cells. Murine I-A^b^ and I-A^d^-restricted polyclonal T cells and T cell clones that were specific for bovine A_1-14_ were shown to require reduction of disulfide bonds for recognition [51]. Human DR1-restricted T cell lines derived from insulin-treated patients were shown to require intact disulfide bonds at A_6_ and A_7_ [52].

### 2.2. CD8^+^ T Cell Responses to Proinsulin

In human T1D, a number of proinsulin epitopes that are presented by MHC class I alleles have been characterized (Table 3). In a first study using peptide library-mediated *in vitro* assembly of class I molecules, proinsulin peptides have been defined on the basis of their association with HLA-B8, A2, and B15. Several epitopes harbor anchor residues that were only weakly predicted or not predicted by common algorithms or that did not contain canonical allele-specific binding motifs [53]. Preproinsulin epitopes that carry C-terminal residues that are generated by proteasome digestion *in vitro* follow SYFPEITHI and BIMAS algorithm prediction and bind *in vitro* to purified class II allele have been further characterized [54–56]. In case of the common HLA-A*0201 allele, immunogenicity in class I knock out A2.1 transgenic mice has further been evidenced [54, 56]. However, self-tolerance to mouse proinsulin epitopes is expected to interfere with immunogenicity of human proinsulin peptides in these mice. CTL that could be maintained *in vitro* after restimulation were cytotoxic to A2.1 target cells, indicating that corresponding proinsulin epitopes were naturally processed by cells expressing proinsulin. Further studies characterized selected peptides within the proinsulin B-C region for recognition by peripheral blood mononuclear cells from A1, A2, A3, A11, A24, B8, and B18 type 1 diabetic patients [55] and peptides located within the preproinsulin leader sequence [56, 57]. T cells specific for leader sequence peptide_15-24_ were shown cytotoxic to human islets expressing HLA-A*0201, bringing further evidence that corresponding T cells may participate to *β*-cell destruction along the human disease process [57]. Leader sequence peptide_14-23_ has been shown distinct from peptide_15-24_ [56], but peptide_15-24_ has been eluted from HLA-A*0201 molecules [57]. A majority of T1D patients shows significant responses to at least one of the peptides covering the whole preproinsulin sequence, while no response is usually observed in control individuals, including type 2 diabetic patients who are treated with exogenous insulin. There is no correlation between the prevalence of responses to proinsulin peptides and the affinity levels of peptide binding to purified HLA class I molecules. In many patients, responses are observed to several peptides. However, the long preclinical phase that precedes clinical diabetes does not preclude that a more restricted set of peptides is recognized at initiation of the autoimmune process. More surprisingly, proinsulin peptides were recognized both in recent-onset and long-standing diabetic patients [55, 56]. This may indicate that long-term memory class I-restricted T cells persist in the long term range in patients who have been deprived of residual *β* cells for years. Long term persistence of memory CD8^+^ T cells may explain the dramatic recurrence of T1D in recipients of hemigrafts from monozygotic, diabetes-discordant twins [15]. Reactivity to one of the B chain peptides identified, preproinsulin_33-47_/B_10-18_, was shown to elicit a CD8^+^ T cell response in long-standing T1D patients who undergo islet graft rejection using an ELISpot for granzyme, interferon *γ* and IL-10 production and immunostaining with A2.1-peptide tetramers [58]. In the NOD mouse in which no T cell activation is seen in mice deprived of *β*-cells at an early stage of the autoimmune process, full activation of T cells is seen in the absence of residual *β*-cells once the autoimmune process initiated [59]. The frequency of preproinsulin-specific CD8^+^ T cells has been estimated, using interferon *γ* ELISpot assays, at a median frequency of 0.004% (range 0.0008–0.08%) of PMBCs [32]. In this last study, they waned within 6 months after diabetes onset. However, persistence of CD8^+^ T cell responses have been observed in long-standing, insulin-treated T1D patients, in particular to B chain peptides, as opposed to leader sequence peptides [55, 56]. The combination of high sensitivity flow cytometry detection and multiplex fluorescent reagents [60] is likely to allow high throughput CD8^+^ T cell analyses complemented by functional studies in a near future.

## 3. T Cell Recognition of GAD, IA-2, and IGRP

While insulin can be defined as a primary target of the autoimmune response to *β*-cells in the NOD mouse, direct evidence is lacking in the human. T cell recognition of autoantigens other than insulin has been defined in the human. The lack of routine human T-cell assays is due to intrinsic difficulties in measuring T-cell responses, in particular in case of CD4^+^ T cells [61]. Circulating antigen-specific T cells are present at a very low frequency. Although these cells are sometimes detectable *ex vivo*, their rarity challenges the sensitivity of technologies such as enzyme-linked immunospot (ELISpot) and flow cytometry. Peptides that bind to HLA class II molecules for presentation and recognition by T-cells have been more difficult to characterize in case of CD4^+^ than CD8^+^ T cells, due to looser binding constraints in case of class II than class I epitopes. Humanized mouse models expressing human class I or class II HLA molecules have largely been used to define class I-restricted and class II-restricted epitopes.

### 3.1. CD4^+^ T Cells

Following the discovery of insulin as an autoantigen in T1D, GAD was discovered as a second autoantigen based on homologies of antigen precipitates with the target of autoantibody in stiff man syndrome. IA-2 was then discovered in 1994. Autoantibodies detected in recent-onset T1D patients recognize predominantly the cytoplasmic domain of IA-2, which shares 80% sequence homology with another tyrosine phosphatase, also known as phogrin [62, 63]. Several approaches have been developed to define CD4^+^ T cells in T1D. An extensive description of epitopes recognized has been provided in a previous review [43]. Increased proliferation of CD4^+^ T cells has been reported in the presence of GAD extracted from pig or human brain and islets [64, 65], overlapping peptides covering the GAD65 and GAD67 sequences, in particular a region covering residues 473–555, while other regions were recognized by T cells from both T1D patients and controls [66], recombinant GAD65 and GAD67 [67–69] in up to two third of patients with recent-onset or subjects at risk for T1D. However, proliferative responses have also been observed in normal individuals. An inverse relation between the detection of anti-GAD autoantibodies and proliferative responses to GAD has been documented [69]. Proliferative responses to a GAD region located at positions 247–279 has been correlated with responses to residues 32–47 of the coxsackie P2-C viral sequence [70]. It is noteworthy that this region is located outside of the human GAD65 region (GAD_473-543_) that was shown to be immunodominant in T1D patients [66]. T cell proliferative responses were also searched against selected GAD peptides, such as GAD65_506-518_ which shows striking homology with proinsulin_24-36_ [33, 71] or GAD65_247-279_, a response that was correlated to T cell proliferative responses to coxsackie viral peptide P2-C_32-47_ [70]. DQ-restricted responses to recombinant GAD have further been defined in patients [42]. Several publications have since reported proliferative responses of CD4^+^ T cells from T1D patients or autoantibody-positive subjects at risk for T1D in the presence of IA-2 or IA-2 peptides [40, 72]. Using IFN-*γ*/IL-4 double-color ELISPOT, IA-2-specific, interferon *γ*-secreting PMBCs were detected *ex vivo* in T1D patients while not in controls [73]. A dominant IA-2_805-820_ epitope in these studies was shown to share 56% identity with a VP7 rotavirus protein [72]. However, difficulties to develop reliable assays in these pioneering studies have hampered further studies and may have explained variable outcomes, low reproducibility of data, difficult discrimination of responses in T1D patients and controls and failure to establish clinically relevant assays. Standardization of conditions of antigen presentation, restriction by class II HLA molecules and profiles of cytokines produced along these assays were usually unknown in these studies.

The use of transgenic mice expressing functional MHC class II molecules have been useful in helping to characterize GAD and IA-2 class II epitopes. The first class II transgenic mice to be developed carried a DRB1*0401 susceptibility allele. They led to identification of two peptides, selected among overlapping 20 mer peptides as binding to DRB1*0401, as immunogenic and naturally processed by DRB1*0401-expressing mouse spleen cells, GAD65_274-286_ and GAD65_115-127_ [74]. Another study in DR4-transgenic mice found that GAD65_274-286_ and GAD65_115-127_ and an additional peptide, GAD65_551-565_, were immunodominant [75]. GAD65_551-565_ was shown to be naturally processed by using a combination of chromatography and mass spectrometry of peptides bound by HLA-DR401 molecules [76]. However, the class II susceptibility molecule that confers the highest susceptibility in T1D is the HLA-DQA1*0301/DQB1*0302 (DQ8) dimer. Humanized class II mice devoid of endogenous mouse class II genes and expressing DQ8 have been used to characterize autoantigen epitopes presented by DQ8. GAD_247-266_ which shows homology with coxsackie P2-C, and GAD_509-528_-specific, DQ8-restricted Th1 CD4^+^ T cell lines were shown to induce insulitis after adoptive transfer into DQ8-expressing mice treated with a very low dose of streptozotocin [77]. Notably, there are two amino acid differences in GAD_247-266_ (GAD_252_ and GAD_256_) and one amino acid difference in GAD_509-528_ (GAD_509_) between the murine and the human sequence. A strong CD4^+^ T cell response was observed and human GAD65 epitopes (GAD_497-517_, GAD_527-547_, GAD_537-557_) identified using a pool of 19 20–23 mers overlapping peptides spanning two large regions of GAD 65 in transgenic mice expressing DQ8 and backcrossed onto the NOD background for two generations [78]. 

Following identification of GAD65_551-565_ as naturally processed and as eliciting a T cell response in recent-onset T1D patients and individuals at risks [73], soluble HLA-DR401 or -DR404 TMrs complexed to GAD65_551-565_ were used to analyze circulating T-cells from recent-onset T1D patients and at-risk subjects. This allowed detecting high avidity CD4^high^ tetramer-positive cells after expansion *in vitro* and activation on specific plate-bound class II-peptide monomers [79]. Seemingly, expansions were detected in the presence of GAD65_555-567_ GAD65_274-286_ peptides, and proinsulin_B24-C36_, using TMrs_._ [80]. Generating a panel of GAD65-specific T cell lines from HLA-DR*0301/*0401 recent-onset T1D patients, epitopes have also been identified. Two were presented by DR4-expressing APCs, one covering amino acid residues 270–283, in close proximity, but outside the homology region shared with Coxsackie virus P2-C protein, a second covering residues 556–575, the peptide largely overlapping with a 20 mer peptide having the highest affinity to DRB1*0401 among known GAD65 peptides. Both were characterized in two T1D patients carrying the high susceptibility HLA-DR*0401/DQB1*0302 haplotype. Two epitopes (GAD_146-165_ and GAD_174-185_) were presented by APCs expressing the susceptibility HLA-DR*1601 allele, but one (GAD_206-225_) was presented by APCs expressing the resistance allele HLA-DR*1501 [81].

Among the many GAD epitopes characterized, GAD_555-567_ has led to extensive studies, both *ex vivo* and in transgenic mice. The transfer of a DR4-restricted GAD_555-567_-specific CD4^+^ T cell clone induces insulitis in Rag^−/−^I-A^b-/-^B6 DR4 transgenic mice [82] while CD4^+^ T cells carrying TCR transgenes from two distinct GAD_555-567_-specific CD4 T cell clones in B6 DR4-transgenic mice remained tolerant, although through different mechanisms [83]. An increase in the avidity of CD4^+^ T cell recognition of GAD_555-567_ has been reported in three prediabetic subjects along progression from autoantibody positivity to clinical T1D [84].

In addition to studies of direct interactions of CD4^+^ T cells and class II-restricted peptides, cellular binding assays have been used for studying peptide-class II interactions for a large number of DR and DQ molecules. A clustering of peptides has been identified in the COOH-terminal region of GAD and promiscuous peptides have been identified. Most peptides were further shown to bind both diabetes-predisposing and diabetes-protective class II molecules [85–87] and DR as well as DQ molecules [88]. However, limitations in these studies rely with the likelihood that, in contrast with antiviral responses, peptides recognized along the autoimmune response to *β*-cells cannot be predicted on an affinity basis [55, 56], many peptides showing low to medium affinity being recognized and inducing immunogenic responses in humanized mice as recently shown in a DQ8 NOD transgenic mouse [89].

Similar strategies have been followed to study CD4^+^ T cell recognition of IA-2 and IGRP. Using libraries of synthetic peptides overlapping the intracytoplasmic domain of IA-2, a dominant epitope recognized by DR4-restricted T cells from subjects at risk for T1D has first been identified, IA-2_805-820_, which has 100% similarity with a sequence of the rotavirus VP7 protein [90]. Studying a panel of naturally processed islet epitopes by elution from APCs bearing HLA-DR4, IA-2_652-80_, IA-2_709-35_, IA-2_752-75_, IA-2_793-817_, IA-2_853-72_, IA-2_955-76_-specific CD4^+^ T cells have been identified as proinflammatory T cells. Interestingly in this study, the majority of nondiabetic, HLA-matched controls also showed a response against islet peptides, but with the phenotype profile of IL-10-secreting T cells [48]. Two phogrin DQ8 epitopes (ICA512_640-659_ and ICA512_755-776_) previously defined as recognized by diabetogenic T cells in the NOD mouse has been further identified using transgenic mice expressing DQ8 on the NOD background [91]. The evidence for molecular mimicry between an IA-2 (and GAD) epitope and the rotavirus VP7 protein has been further detailed by showing strong binding of both autoantigen and viral peptides to HLA-DRB1*04 and cross recognition by IA-2-specific T cells [92]. The 831–860 region of IA-2 frequently recognized by autoantibodies has been shown to be recognized by IL-10-secreting T cells from T1D patients [93]. Following identification of DRA1*0101/DRB1*0401-retricted IGRP_23-35_ and IGRP_247-259_ and DRA1*0101/DRB1*0301-retricted IGRP_13-25_ and IGRP_226-238_ epitopes, IGRP-specific CD4^+^ T cells have been detected in more than 80% of DRB1*0401 or DRB1*0301 healthy and T1D subjects [94].

Interestingly, autoantigen-specific CD4^+^ T cells have been studied in very different clinical settings, including in autoantibody-positive individuals at risk for T1D, in recent-onset T1D patients and in patients undergoing pancreas/kidney as recently reported in 3 patients [95] or islet transplant [58]. Autoantibodies were detected either pretransplant or reappeared 5 and 6 years posttransplant in still normoglycemic patients, somewhat paralleling insulitis, whatever the immunosuppression used. GAD-specific CD4^+^ T cells were detected using DRB1*0405 and DRB4*0101 TMrs and IGRP-specific CD8^+^ T-cells were detected using HLA-A2/A*0201 class I pentamers along followup. Autoreactive T-cells are temporarily inhibited by immunosuppression, their reappearance is followed by further loss of insulin secretion [95].

### 3.2. CD8^+^ T Cells

The first evidence for the recognition of GAD by A*0201-restricted CD8^+^ T cells was obtained in one asymptomatic and two recent diabetic patients. CD8^+^ T cells detected in this study were shown to target HLA-A*0201 peptide GAD_114-123_ and were cytotoxic to autologous antigen-presenting cells incubated with the GAD_114-123_ peptide or infected with a recombinant vaccinia virus expressing GAD65 [38]. A list of epitopes along with class I-restricted HLA molecule is provided in Table 4. The recognition of the GAD_114-123 _ epitope was confirmed by another study using an interferon *γ* Elispot assay [96]. Another peptide from IA-2 (IA-2_797-805_) was reported as the target of cytotoxic T cells, but both in T1D patients and control individuals [97]. Using algorithms to predict nonameric *β*-cell peptides that would bind to the common HLA-A*0201 allele and an interferon *γ* Elispot assay, a human islet amyloid polypeptide (IAPP) precursor protein, 6 out of 9 recent-onset T1D patients, but none of longstanding T1D patients, were shown to recognize preproIAPP peptide IAPP_5-13 _[98]. Another IAPP peptide (IAPP_9-17_) was defined using the same approach and an assay evaluating granzyme B secretion, along with IGRP peptides IGRP_215-223_, IGRP_152-160_, IGRP_228-236_ and IGRP_266-273_ glial fibrillary acidic protein (GFAP) peptides GFAP_143-151 _and GFAP_214-222_, IA-2_172-180_, and IA-2_482-490_, [95, 99, 100]. A strong inverse correlation between the binding affinity of *β*-cell peptides to HLA-A*0201 and CTL responses against those peptides was observed in recent-onset type 1 diabetic patients. These data confirmed that many *β*-cell epitopes are recognized by CTLs in recent-onset type 1 diabetic patients. Interestingly, IA-2 and GAD have been defined as a key autoantigen in T1D on the basis of the predictive value of anti-IA2 and anti-GAD autoantibodies in prediabetic individuals, while IGRP and IAPP have not been defined as key targets of autoantibodies in T1D in the human. GAD_114-122_-specific CD8^+^ T cells, as well as GAD-specific and insulin-specific CD4^+^ T cells, have further been detected exclusively in T1D patients within the memory CD45RO^+^ T cell population while naïve CD45RO T cell stained with HLA-0201*-GAD_114-122_ tetramers were discriminative between control and T1D patients [101]. A combinatorial quantum dot MHC multimer technique has further allowed detecting expansions of HLA-A*0201-restricted CD8^+^ T cells that were specific for IA-2_797-805_, GAD65_114-123_, IGRP_265-273_, and preproIAPP_5-13_ in recent onset diabetes patients, with a specificity ranging from 87% to 100% and a sensitivity ranging from 25% to 40%, and in islet transplantation recipients [60].

The use of HLA-A*0201 transgenic mice has first reported or confirmed the characterization of class I-restricted peptides that are potentially presented to CD8^+^ T cells in the human. IGRP_206-214_, IGRP_337-345_ and IGRP_265-273_ have been identified in HLA-A*0201 transgenic mice on the NOD genetic background and shown to be targeted by pathogenic CD8^+^ T cells [102]. The systematic immunization of HLA-A*0201 transgenic mice using plasmids encoding GAD65 or the catalytic unit of the intracellular domain of IA-2 has allowed defining 5 GAD peptides (GAD_110-118_, GAD_114-123_, GAD_159-167_, GAD_476-484_, and GAD_536-545_) and 4 IA-2 peptides (IA2_790-798_, IA2_805-813_, IA2_830-839_, and IA2_962-970_) that were recognized by CD8^+^ T cells from T1D patients, 3 of which (GAD_114-123_, GAD_536-545_, and IA2_805-813_) in more than 25% patients [103].

Shifts both in frequency and in immunodominance of CD8^+^ T-cell responses have been observed within months following T1D onset and were more rapid than changes in autoantibody titers. Positive T-cell responses to islet epitopes (GAD65_114-123_, GAD65_536-545_, IGRP_228-236_, PPI_2-10_, PPI_34-42_, PPI_42-51_, and PI_101-109_) observed at diagnosis were shown to drop to non detectable levels, while newly targeted epitopes were evidenced, in particular proinsulin B_18-27_, IA-2_206-214_, and IGRP_265-273_. However, of a total of positive T-cell responses to islet epitopes observed at diagnosis, 26 of 42 dropped to nondetectable levels, while new epitopes were targeted in only 5 [32].

### 3.3. Regulatory T Cells

The role of protective CD4^+^ T cells in T1D has first been defined in the NOD mouse [104]. In the human, initial reports documenting decreased numbers of CD4^+^ T regulatory (T_reg_) cells defined as CD25^+^ [105] were not confirmed by later works using more specific surface phenotyping [106]. Few papers addressed the issue of T_reg_ by analyzing autoantigen-specific T_reg_. Despite the importance of this question, the characterization of islet-specific Tregs is still in its infancy, as it is rather difficult to detect them (their frequency being probably even lower than that of the corresponding effectors) and to expand them *in vitro* (most of these cells being characterized by a state of anergy that needs to be reversed).

The characterization of both proinflammatory (IFN-*γ*-producing) and regulatory (IL-10-producing) CD4^+^ T-cell responses against proinsulin and IA-2 indicate that T_reg_ are possibly key players. While T1D patients harbored predominant IFN-*γ* responses, healthy subjects were characterized by higher frequencies of IL-10 responses specific for the same epitopes. The same was not true for responses against an irrelevant Ag such as tetanus toxoid. Moreover, T1D patients displaying higher IL-10 responses were characterized by an older age T1D onset, suggesting that these regulatory may counterbalance autoimmune effectors, at least transiently [48]. IL-10-secreting CD4^+^ T_reg_ specific for proinsulin and IA-2 epitopes have been characterized as suppressive *in vitro *in healthy subjects. This suppressive activity is however not linked to IL-10 secretion, but rather to elimination of Ag-presenting cells [107]. Importantly, we previously showed that GAD-specific effector CD4^+^ T cells cloned from T1D patients could also be rendered anergic and suppressive upon sustained Ag-specific *in vitro* stimulation [108]. Similar observations were made by showing that patients harboring the protective I/III and III/III insulin VNTR haplotypes displayed a threefold higher IL-10 release in proinsulin-specific memory T cells. These data are consistent with the hypothesis that VNTR-induced higher insulin levels in the thymus promote Treg generation, offering an additional explanation for the protective effect of the VNTR class III alleles [109]. Seemingly, cloned PPI_70-90_-specific DRB1*0401-restricted human T cells have been characterized as expressing a downregulatory T helper 2 phenotype through predominant production of IL-5 and IL-10 and low interferon *γ* production [110]. DRB1*0401-restricted CD4^+^ T cells that are specific for GAD65_555-567_ have been identified in normal individuals using GAD65-specific class II TMrs after expansion in the presence of peptide, following removal of CD4^+^CD25^+^T_reg_. Their expansion was reversed adding back CD4^+^CD25^+^T_reg_ [111, 112]. Taken together, these observations suggest that detection of T-cell autoreactivity should not be considered pathological *per se*, but should rather be interpreted by functional profiling. Regulatory PI- and GAD-specific T-cell clones can be obtained *in vitro* under standard stimulating conditions in the absence of any exogenous cytokines [113]. Similarly, GAD- and IGRP-specific CD4^+^ Treg clones can be obtained by *in vitro* stimulation of FoxP3-negative CD4^+^ T cells [114]. Whether such clones are only generated *in vitro* or can also be isolated *ex vivo* and their mechanism(s) of suppression remain important questions for further investigation. On the same line, there have been reports indicating that GAD-specific circulating CD4^+^ T cells show an activation phenotype that is not seen in control individuals, likely a memory phenotype and are more prone to proliferate while less dependent on CD28/B7-1 costimulation [115]. GAD65- and proinsulin-specific T-cells, including cells that were specific for GAD_106-125_, GAD_526-545_, GAD_266-285_, GAD_556-575_, hPPI_72-90_ and hPPI_94-110_, have been shown to coexpress CD25 and CD134 (OX40) as a distinctive feature when compared to T cells from healthy subjects [116]. Beyond, CD4^+^ T cells, polyclonal regulatory CD8^+^ T cells have also been characterized. CD8^+^ CD45RA^+^ CD27^−^ T cells have been shown to control GAD65-specific CD4^+^ T cell expansions through a contact dependent mechanism and the production of IL-10 [117].

## 4. Therapeutic Trials Using Peptides or Autoantigens in the Human

The characterization of T-cell epitopes is expected to help developing T cell assays to be used in the followup of immunotherapy trials in T1D, thus providing surrogate end point markers of tolerance induction that may prove more reliable than current autoantibody assays [118, 119] and pave the way towards antigen or peptide-specific immunotherapy. 

### 4.1. Mechanisms of Tolerance

A major advantage of antigen or peptide-specific immunotherapy over other forms of immunotherapy in human autoimmune diseases is in focusing treatment on self-reactive T cell clones without impairing immune responses to unrelated antigens, especially tumoral or infectious antigens. In T1D in particular, this may prove of outmost importance considering that insulin therapy has gained in efficacy and safety over years, explaining the progressively decreasing mortality gap between T1D patients and the general population [120]. Although nonspecific immune suppression has been shown partially effective in preserving *β*-cells from autoimmune destruction in recent-onset T1D patients, immunosuppressive drugs used to suppress the immune response have shown major side effects that preclude their use in the long-term range. Peptide and/or antigen-specific immunotherapy is thus likely to allow optimal risk/benefit ratio in T1D.

Immune tolerance is ensured in a succession of checkpoints by a variety of mechanisms affecting differentiating lymphocytes in central lymphoid organs as well as mature lymphocytes in the periphery. Tolerance in the periphery rely on deletion of autoreactive cells, on ignorance of self antigens, on active mechanisms that imprint an intrinsic status on tolerant lymphocytes in the form of anergy or immune deviation and on extrinsic mechanisms that involve regulatory cells. Molecular interactions in the presentation of autoantigen in the periphery are central to the tolerance process and in strategies aiming at restoring or inducing immune tolerance in autoimmunity. Elimination or reprogramming of deleterious autoreactive cells and activation of regulatory cells to control autoimmune effectors are the major outcomes expected from antigen or peptide-specific immunotherapy. These mechanisms have been clearly documented in preclinical models but are only starting to be implemented in human studies.

Key advantages of inducing antigen-specific immune tolerance have been underscored but depend on mechanisms of tolerance induction. First, it may not require knowing the initiating target autoantigen, nor the fine specificity of T cells involved. Induction of regulatory T cells, whatever their specificity, may induce bystander immunomodulation within inflammatory sites, for instance, through *in situ* production of protective cytokines, spreading of Th2 responses or promoting tolerizing antigen presentation. Factors affecting the efficiency of antigen-based immunotherapy include the size of the antigen used, the autoantigen expression pattern, the stage of the disease process at time of administering the tolerizing autoantigen, the crypticity of the epitopes presented, the autoantigen administration route and dose [121].

Given the molecular constraints of T cell activation, the induction of peptide-specific tolerance is expected to require presentation of specific peptides to autoreactive CD4^+^ T cells in a noninflammatory environment. Induction of immune tolerance by injection of high doses of soluble peptide or antigen or DNA vaccination has proven efficient in experimental models of autoimmunity, but concerns have been raised by the risk of either exacerbating the autoimmune process in some experimental conditions or inducing anaphylactic reactions. The induction of tolerance by the mucosal (oral or nasal) route has seemingly been shown efficient, mostly in preventing autoimmunity, in preclinical models but has failed to apply to human diseases. Mechanisms of action of the mucosal route differ depending on the dose of antigen delivered. The dose chosen in human trials has so far been random. A promising approach to induce tolerance in autoimmune diseases is intravenous injection of antigen-coupled, ethylene carbodiimide-(ECDI-) fixed splenocytes. It has shown efficient in animal models, but is more complex to set up in the human. Among mechanisms involved, both the induction of anergy, at least in part through suboptimal costimulatory signaling, and presentation of the tolerizing epitope by plasmacytoid dendritic cells have been evidenced. As a last example, altered peptide ligands (APLs), either antagonistic or partial agonist APLs, have been successfully used in preclinical models to prevent autoimmunity through anergy, immune deviation or bystander suppression. As in case of soluble peptide or antigen, the use of APLs has raised safety issues relating with either exacerbation of autoimmunity or anaphylactic reactions in the human in multiple sclerosis [122]. Deviation of autoimmunity to different targets is another danger that should not be excluded [1, 123, 124].

### 4.2. Insulin Trials

As a key autoantigen in T1D, insulin has been used in several trials to downregulate the autoimmune response in recent onset patients or to prevent the full destruction of *β*-cells in prediabetic subjects. The first trial ever to modulate *β*-cell destruction used intravenous insulin delivered by an external artificial pancreas to maintain glycemic values between 3.3 and 4.4 mmol/L during 14 days at onset of T1D. This study in 12 T1D patients was compared to conventional treatment using subcutaneous NPH insulin injections (*n* = 14) and showed significantly higher C-peptide values in the experimental as compared to the conventional group at one-year. Insulin doses in the experimental group were in the order of 3 U/kg/d. Mechanisms may have involved *β*-cell rest as well immunomodulation through intravenous delivery of insulin [125]. It is possible that efficient metabolic control rather than high insulin doses explain the preservation of *β* cell function in this study. In a comparable study in recent-onset T1D, the nine patients who received high-dose intravenous insulin infusion and the ten patients under intensive-therapy group equally preserved *β* cell function along a one year follow-up [126]. By contrast, in the DCCT study, intensive insulin therapy allowed maintaining higher C-peptide levels than conventional treatment with one or two injections a day [127]. Fifteen years later, the first randomized, double-blind crossover study using nasal delivery of insulin in 38 prediabetic, autoantibody-positive individuals showed an increase in anti-insulin antibodies and a decrease in T cell proliferative responses to insulin, while no acceleration of diabetes development and stable first-phase insulin response to glucose in the 26 individuals who did not develop diabetes was observed at one year [128]. The Diabetes Prevention Trial-Type 1 Diabetes Study [129] screened 84 228 first and second-degree relatives of T1D patients to select 3152 autoantibody-positive individuals and assigned 339 with a projected five year risk over 50% to close observation or low-dose subcutaneous ultralente insulin, 0.25 U/kg/d plus annual 4 day courses of continuous intravenous insulin infusions with no delay in diabetes development after a median followup of 3.7 years. In a group of individuals with a five year projected risk of 26%–50%, no significant difference in the development of T1D was observed between individuals randomly assigned to oral insulin *versus *individuals assigned to placebo. However, in a subgroup of individuals with anti-insulin autoantibodies ≥80 nU/mL, 6.2% individuals receiving oral insulin developed T1D, as compared to 10.4% of those receiving placebo (*P* < .015), suggesting that oral insulin should be tested specifically in this subgroup of individuals [130]. However, a more recent, double-blind, randomised, controlled study in 115 individuals receiving intranasal insulin (1 U/kg/d) *versus* 109 infants and siblings with ≥2 autoantibodies showed no difference between the two groups [131]. It is even possible in this study that nasal insulin had an accelerating effect on T1D development in individuals with ≥3 autoantibodies against different antigen specificities. These data face major caveats. First, the dose of insulin delivered has little rationale. Second, it is likely that prediabetes corresponds to fully activated autoimmunity involving an already expanded T cell repertoire. In new-onset T1D patients, oral delivery of human insulin at doses ranging from 2.5 to 7.5 mg/d failed to show any benefit on C-peptide secretion at one year [132, 133]. Again, new-onset T1D is likely a late stage hardly accessible to down regulation of the autoimmune reaction. Administration of insulin B-chain in incomplete Freund's adjuvant has been shown to elicit strong B and T cell immune responses to insulin in 12 subjects with recent-onset T1D, but no C-peptide benefit over a two-year followup [134]. Finally, beyond the use of insulin as a key autoantigen to downregulate the autoimmune response to *β*-cells in prediabetes and in new-onset T1D, a key issue remains whether or not immediate insulin therapy should be started in noninsulin dependent diabetes patients who are tested positive for islet cell autoantibodies, in particular anti-GAD autoantibodies. Sixty anti-GAD positive patients with a five year non-insulin dependent diabetes profile were randomized to either early insulin or sulfonylureas, tested annually for C-peptide secretion under an oral glucose tolerance test and followed up for 57 months. The progression to insulin dependence was observed in 3 out of 30 patients in the insulin group while in 13 of 30 in the sulfonylurea group [135], in contrast with other studies [136].

### 4.3. Glutamate Decarboxylase Trials

Following a dose-finding study in patients with latent autoimmune diabetes in adults indicating the safety of a primary injection and a booster injection of 20 *μ*g each of recombinant human GAD in a standard vaccine formulation with alum as adjuvant [137], a double-blind randomised study was performed in 35 GAD autoantibody-positive T1D patients who had fasting C-peptide levels >0.1 nmol/L (0.3 ng/mL) within 18 months of diabetes diagnosis. Patients in the treated group received two injections of GAD at 1 and 30 days of the study. The decrease in fasting C-peptide values and stimulated secretion with time was significantly lower by 23% and 29%, respectively, in treated *versus* placebo patients, 30 months after the first GAD injection. No adverse effects were observed. An increase in anti-GAD autoantibodies was seen in patients who received GAD injections and peaked at 3 months of the study. Interleukins 5, 10, 13, and 17, interferon-*γ* and TNF-*α* production by T cells in response to GAD *in vitro* were seen in treated patients, while not in the placebo group [138].

### 4.4. Heat Shock Protein Trials

Heat shock protein 60 (hsp60) has been discussed following NOD studies as an autoantigen in T1D and T cell clones specific for hsp60 peptide p277 characterized in this model. Several trials have been conducted in the human using a 24-aminoacid peptide derived from the C-terminus of human hsp60 in patients with established T1D, including a trial in children with T1D. While no effect on C-peptide preservation was observed in the pediatric study, one of the randomized study performed in 35 adult T1D patients within <6 months following diagnosis reported significant preservation of C-peptide values in the treated group as opposed to significant decrease in placebo-treated patients. T-cell responses showed increased interferon *γ* and decreased interleukin 13 production in response to hsp60 than to p277 and less interferon *γ*, more interleukin 10 and 13 in the treated group than in the placebo group [139].

## 5. Conclusion

Developing T-cell assays that allow characterizing diabetes autoimmunity is a major challenge in human T1D. It is expected to help defining epitopes that are recognized in T1D, to pinpoint key functional features of epitope-specific T lymphocytes along the natural history of the diabetes process and to pave the way towards therapeutic strategies to induce immune tolerance to *β*-cells. New T-cell technologies are expected to allow defining autoreactive T-cell differentiation programs and characterizing autoimmune responses in comparison to physiologically appropriate immune responses. This may allow additional mechanistic studies and prove instrumental in the discovery of immune correlates of efficacy in clinical trials, as initially reported using autoantibody assays and now an open field for T cell assays.

## Figures and Tables

**Table 1:** Autoantigens defined as recognized by T cells in human T1D. Listing has been limited to autoantigens for which evidence of recognition has been obtained in the human or, if only in the mouse, data are expected in the human.

autoantigen	expression	Subcellular location	Involvement in the NOD mouse	Human T1D		
				autoantibodies	CD4^+^ T cells	CD8^+^ T cells
Insulin	*β*-cell, thymus	secretory granule	+	+	+	+
*GAD 65	neuroendocrine	synaptic-like microvesicles	+	+	+	+
GAD 67	neuroendocrine	cytosol	+	+	+	+
IA-2 (ICA512)	neuroendocrine	secretory granule		+	+	+
IA-2 *β*/phogrin	neuroendocrine	secretory granule		+	+	+
IGRP	*β*-cell	endoplasmic reticulum	+	?	+	+
Chromogranin	neuroendocrine	Secretory granule	+	?	?	?
ZnT8	*β*-cell	secretory granule	?	+	?	?
HSP-60 HSP-70	ubiquitous	mitochondria	+	+	+	?
Glima-38		secretory granule	?	+	?	?
Amylin/IAPP		secretory granule	?	?	?	+
CD38	ubiquitous	?	?	±	?	?

GAD: glutamate decarboxylase; IA-2: islet antigen 2; ZnT8; HSP: heat shock protein; IAPP; IGRP; ICA: islet cell antibody.

?: no positive results reported.

**Table 2:** Class II-restricted* CD4^+^ T-cell epitopes on human preproinsulin.

^§^Epitope preproinsulin	^§^Epitope Insulin nomenclature	MHC restriction	responders	references
PPI_1-24_	L_1-24_	DQ8	Transgenic mice	[44]
PPI_11-26_	L_11_-B_2_	DRB1*0401	Transgenic mice	[45]
PPI_14-33_	L_14_-B_9_	DQ6	Transgenic mice	[44]
PPI_20-36_	L_20_-B_12_	DRB1*0401	Transgenic mice	[45]
PPI_21-36_	L_21_-B_12_	DR4	Transgenic mice	[36, 45]
PPI_33-47_	B_9-23_	DQ8	At risk/recent-onset Transgenic mice	[35, 47]
PPI_35-51_	B_11-27_	DR16	At risk	[36]
PPI_44-63_	B_20_-C_7_	DQ8	Transgenic mice	[44]
PPI_59-74_	C_3_-C_18_	DR	Human T cell lines	[140]
PPI_73-90_	C_17_-A_1_	DR4	Transgenic mice	[45]
PPI_75-91_	C_19_-A_3_	DR4	T1D	[48]
PPI_78-94_	C_22_-A_5_	DR4	T1D	[48]
PPI_74-93_	C_19_-A_4_	DQ6	Transgenic mice	[44]
PPI_70-93_	C_13_-A_6_	DRB1*0401	Transgenic mice	[45]
PPI_85-101_	C_29_-A_12_	DRB1*0401	Transgenic mice	[45]
PPI_69-88_	C_13-32_	DR4	T1D	[48]
PPI_75-92_	C_19_-A_3_	DR4	T1D	[48]
PPI_78-94_	C_22_-A_5_	DR4	T1D	[48]
PPI_27-102_	C_29_-A_12_	DR4	Transgenic mice	[45]
PPI_90-104_	A_1-15_, A_1-13_	DRB1*0401	T cell clones	[49, 51]
PPI_90-102_			Long-standing T1D	

^§^The preproinsulin nomenclature here refers to the human preproinsulin sequence (errors in some publications have been corrected here, which explains differences with cited references):

leader sequence: MALWMRLLPLLALLALWGPDPAAA;

B chain: FVNQHLCGSHLVEALYLVCGERGFFYTPKT;

C peptide: (RR)EAEDLQVGQVELGGGPGASGLQPLALEGSLQ(RR), (R) are excised during insulin processing;

A chain: GIVEQCCTSICSLYQLENYCN.

^§§^Epitopes for which class II-restriting alleles have not been defined are not indicated in this table: PPI_1-16_ (L_1-16_), PPI_5-20_ (L_5-20_), PPI_9-24_ (L_9-24_), PPI, L_13_-B_4_, L_17_-B_8_, B_1-16_, B_6_–B_22_, B_16-32_, B_25_-C_9_, PPI_67-83_, C_13-29_ [36]; B_1_–B_17_, B_11_–B_27_, B_20_-C_4_, B_24_-C_4_, B_30_-C_14_, C_8_–C_24_, C_18_-A_1_, C_28_-A_11_, A_6_–A_21_ [141]; B_10-25_, B_25_-C_8_, [140–142].

**Table 3:** CD8^+^ T-cell epitopes on human preproinsulin.

Epitope preproinsulin	Epitope Insulin nomenclature	MHC restriction	responders	references
PPI_2-10_	L_2-10_	HLA-A*0201	Recent-onset T1D	[96]
PPI_2-11_	L_2-11_	HLA-A*0201	Recent-onset T1D	[56]
PPI_2-11_	L_2-11_	HLA-A24	Recent-onset T1D	[56]
PPI_2-11_	L_2-11_	HLA-B8	Recent-onset T1D	[56]
PPI_6-14_/PPI_6-16_	L_6-14_/L_6-16_	HLA-A*0201	Recent-onset T1D	[56]
PPI_14-23_	L_14-23_	HLA-A*0201	Recent-onset T1D	[56]
PPI_15-25_	L_14_-B_1_	HLA-A*0201	Recent-onset T1D	[56]
PPI_15-24_	L_15_–L_24_	HLA-A*0201	Recent-onset T1D	[57]
PPI_15-25_	L_14_-B_1_	HLA-A*0201	Transgenic mice	[54]
PPI_23-42_	L_23-18_	HLA-A24	Recent-onset T1D	[56]
PPI_34-42_	B_10-18_	HLA-A*0201	Recent-onset T1D	[54, 55, 58]
			Islet graft rejection	
			Transgenic mice	
PPI_38-46_	B_14-22_	HLA-A3	Recent-onset T1D	[55]
PPI_38-46_	B_14-22_	HLA-A11	Recent-onset T1D	[55]
PPI_39-47_	B_15-23_	HLA-A24	Recent-onset T1D	[141]
PPI_39-48_	B_15-24_	HLA-A24	Recent-onset T1D	[55]
PPI_41-50_	B_17-26_	HLA-A1	Recent-onset T1D	[55]
PPI_41-50_	B_17-26_	HLA-A3	Recent-onset T1D	[55]
PPI_41-50_	B_17-26_	HLA-A11	Recent-onset T1D	[55]
PPI_42-51_	B_18-27_	HLA-A1	Recent-onset T1D	[54, 55]
			Transgenic mice	
PPI_42-51_	B_18-27_	HLA-A*0201	Recent-onset T1D	[55]
PPI_42-51_	B_18-27_	HLA-B8	Recent-onset T1D	[55]
PPI_42-51_	B_18-27_	HLA-B18	Recent-onset T1D	[55]
PPI_44-51_	B_20-27_	HLA-A1	Recent-onset T1D	[55]
PPI_44-51_	B_20-27_	HLA-B8	Recent-onset T1D	[55]
PPI_45-53_	B_21-29_	HLA-A3	Recent-onset T1D	[55]
PPI_49-57_	B_25_-C_1_	HLA-B8	Recent-onset T1D	[55]
PPI_51-61_	B_27_-C_ 5_	HLA-B8	Recent-onset T1D	[55]
PPI_76-84_	C_20-28_	HLA-A*0201	Transgenic mice	[54, 96]
			Recent-onset T1D	
PPI_83-89_	C_27_-C_(34)_	HLA-A*0201	Transgenic mice	[54]
PPI_85-94_	C_29_-A_5_	HLA-A*0201	Transgenic mice	[54, 96]
			Recent-onset T1D	
PPI_90-99_	A_1-10_	HLA-A*0201	Transgenic mice	[54]
PPI_101-109_	A_12-20_	HLA-A*0201	Transgenic mice	[54, 96]
			Recent-onset T1D	

**Table 4:** CD8^+^ T-cell epitopes of human T1D autoantigens (other than preproinsulin).

Epitope preproinsulin	MHC restriction	responders	references
GAD_114-123_	HLA-A*0201	Recent-onset T1D	[38, 96, 103]
		Transgenic mice	
GAD_114-122_	HLA-A*0201	Recent-onset T1D	[101]
GAD_110-118_	HLA-A*0201	Transgenic mice	[103]
GAD_159-167_	HLA-A*0201	Transgenic mice	[103]
GAD_476-484_	HLA-A*0201	Transgenic mice	[103]
GAD_536-545_	HLA-A*0201	Transgenic mice	[103]
IAPP_5-13_	HLA-A*0201	Recent-onset T1D	[98]
IAPP_9-17_	HLA-A*0201	At risk	[99, 100]
		Recent-onset T1D	
		T1D	
IGRP_215-223_	HLA-A*0201	Recent-onset T1D	[100]
IGRP_152-160_	HLA-A*0201	At risk	[99, 100]
		T1D	
IGRP_228-236_	HLA-A*0201	Transgenic mice	[96, 102]
		Recent-onset T1D	
IGRP_266-273_	HLA-A*0201	Transgenic mice	[96, 102]
		Recent-onset T1D	
IGRP_206-214_	HLA-A*0201	Transgenic mice	[102]
IGRP_337-345_	HLA-A*0201	Transgenic mice	[102]
IGRP_265-273_	HLA-A*0201	Transgenic mice	[102]
GFAP_143-151_	HLA-A*0201	At risk	[99]
		T1D	
GFAP_214-222_	HLA-A*0201	At risk	[99]
		T1D	
IA-2_797-805_		Normal subjects	[97]
		Recent-onset T1D	
IA-2_172-180_	HLA-A*0201	Recent-onset T1D	[100]
IA-2_482-490_	HLA-A*0201	Recent-onset T1D	[100]
IA2_790-798_	HLA-A*0201	Transgenic mice	[103]
IA2_805-813_	HLA-A*0201	Transgenic mice	[103]
IA2_830-839_	HLA-A*0201	Transgenic mice	[103]
IA2_962-970_	HLA-A*0201	Transgenic mice	[103]
